# In Vivo Evaluation of a Novel Long‐Term Intravascular Implantable Continuous Blood Glucose Monitor in an Ovine Model: A Glucotrack Inc. Investigation

**DOI:** 10.1155/jdr/8838426

**Published:** 2026-04-25

**Authors:** Mark A. Tapsak, Michael Talcott, James P. Thrower, Paul V. Goode, Timothy L. Routh, Stephen T. Tapsak, Jose Garcia

**Affiliations:** ^1^ Research and Development, Glucotrack Inc., Front Royal, Virginia, USA; ^2^ Division of Cardiology, Washington University in St. Louis, St. Louis, Missouri, USA, wustl.edu

## Abstract

Implantable intravascular continuous blood glucose monitors (CBGMs) offer a promising approach for accurately measuring glucose levels with short lag times and improved reliability when compared with conventional laboratory‐based point‐of‐care or handheld glucometers. In this study, 34 Glucotrack long‐term intravascular CBGM devices were implanted into 17 adult sheep, with one device placed in each jugular vein. Each implantation was completed in approximately 20 min and did not require any customized tools or techniques. Intravenous glucose tolerance tests (IVGTTs) were performed approximately monthly throughout the studies (30–240 days postimplantation) and mean absolute relative difference (MARD) values were calculated against an Accu‐Chek Guide handheld glucometer as the reference standard. The weighted average MARD was 6.84% for all 34 devices during a total of 79 IVGTTs. In addition, no device‐related adverse safety events were observed during this long‐term in vivo proof‐of‐concept evaluation.

Summary

• **Successful implantation:** Thirty‐four Glucotrack intravascular continuous blood glucose monitors (CBGMs) were implanted in 17 sheep using standard tools and techniques. Each implantation was completed in approximately 20 min. No device‐related complications or adverse safety events were observed over the implant period.
• **Long-term accuracy and stability:** The devices maintained consistent glucose sensing performance up to 240 days. Across 79 intravenous glucose tolerance test (IVGTT) studies performed at 30–240‐day timepoints, the weighted average MARD was 6.84% and average glucose sensitivity remained within a comparable range for the duration of the study.
• **No safety concerns:** No device‐related adverse safety events were clinically evident during the study.
• **Further research potential:** The results support further investigation into long‐term intravascular glucose monitoring.

## 1. Introduction

An estimated 463 million people worldwide have diabetes, and 374 million people have impaired glucose tolerance [1]. The American Diabetes Association (ADA) recommends using continuous glucose monitoring especially for patients with diabetes on insulin therapy [2]. The use of continuous glucose monitors (CGMs) increased from < 1% to 47% in youth and from 2% to 37% in adults with Type 1 diabetes (from 2008–2010 to 2020–2022) over the last 15 years [3, 4]. Recent market trends suggest that about 60% of people with Type 1 diabetes (Close Concerns 4Q23 Industry Roundup, April 15, 2024) and 50% of people with Type 2 diabetes (RBC Capital Markets, Dexcom Analyst Report, March 2024) use a CGM for glucose management. Barriers to CGM adoption and adherence, such as comfort of wear, may be overcome by implantable continuous glucose monitoring devices [5, 6]. An implantable CBGM in the intravascular space is expected to provide stable, real‐time glucose measurements compared with subcutaneous sensors [7–9]. The placement of a CBGM in the intravascular space requires that it must overcome the foreign body reaction and generation of thrombus [10]. Prior attempts to develop an intravascular CGM have found limited success and therefore are not widely available [11] (FDA Devices Database 2024).

**Figure 1:**
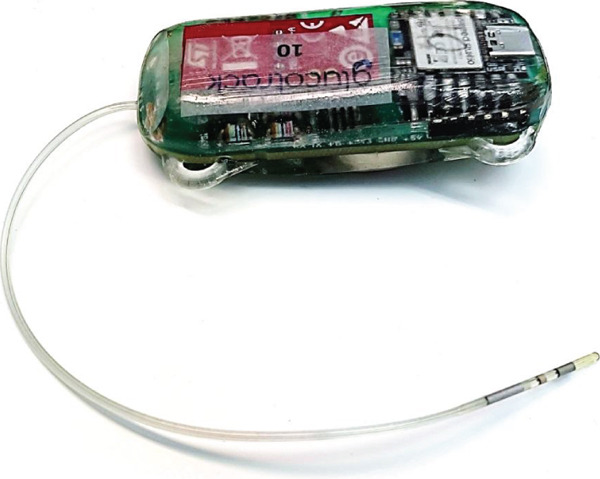
Prototype Glucotrack CBGM device. The Glucotrack CBGM device consists of a device housing and one sensing lead. Minor updates were made to improve device longevity in following device prototypes used in the studies.

Glucotrack′s novel intravascular CBGM is an amperometric glucose oxidase‐based sensor. The CBGM implant prototypes consist of a device housing implanted in the subcutaneous space and a sensing lead implanted in the intravascular space. An example of the initial prototype may be seen in Figure 1. The housing contains electronics, battery, data storage, and wireless communication components (near‐field communication [NFC] or Bluetooth low energy [BLE]). The sensing lead contains a 12‐mm^2^ working electrode (Pt90:Ir10) coated with a well‐established glucose oxidase‐based membrane system, a reference electrode (Ag/AgCl, Pt90:Ir10, or IrOx), and a counter electrode (Pt90:Ir10). The data in the BLE enabled devices were automatically transmitted via mobile phone to a cloud‐based database for real‐time monitoring. In the NFC‐enabled devices, portions of the data were retrieved periodically using an off‐the‐shelf NFC reader to minimize handling and restraining stress on the animals. The full datasets were retrieved from the NFC devices after explantation.

This report presents the first in vivo evaluation of Glucotrack′s prototype intravascular CBGM in a chronic ovine model (up to 240 days). The system demonstrated high reliability during glucose excursions when compared with measurements made using an Accu‐Chek Guide handheld glucometer. The wool and lanolin production of sheep prevented the use of percutaneous CGMs as comparator devices. No device‐ or procedure‐related serious adverse events were observed throughout the studies.

## 2. Research Design and Methods

### 2.1. Ovine Model and Experimental Design

Seventeen adult male Suffolk Crossbred sheep (30–60 kg) were selected for in vivo testing at Pharmaron San Diego Lab Services (Carlsbad, California). Sheep were chosen as the large‐animal model because they offer several physiological and anatomical advantages for evaluating venous glucose‐sensing technologies. Notably, their coagulation cascade is similar to that of humans [12], and the jugular vein provides an excellent analogue to the human subclavian vein in both size and venous flow rates [13]. In addition, sheep typically exhibit glycemic ranges of 30–80 mg/dL [14], making them suitable for assessing sensor performance across the lower end of the CGM sensing range (40–400 mg/dL), including values relevant to human hypoglycemia.

However, despite their physiological suitability, standard transcutaneous CGMs cannot be reliably used in sheep because wool density and lanolin production interfere with sensor adhesion and function. In addition, commercially available CGM systems do not provide raw glucose data to the end user; instead, they deliver filtered and/or time‐lag–compensated values due to the interstitial nature of the measurement. This makes them unsuitable as a reference standard for evaluating a device designed to measure lag‐free intravenous glucose concentrations. For these reasons, blood glucose measurements obtained via finger‐prick handheld glucometers were used as the reference method.

The studies were approved by the Pharmaron Lab Service Institutional Animal Care and Use Committee (IACUC). Animals were fed twice daily on a standard diet of pellets and hay/alfalfa, except during overnight fasting before surgical procedures or glucose excursions. Animals had ad libitum access to water. Body weights were recorded weekly. Animals were observed for any signs indicating moribund condition, including impaired ambulation preventing access to food or water, emaciation or excessive weight loss (> 20%), reduced physical or mental alertness, labored breathing, or inability to remain upright. Pain, distress, or any other clinical signs were assessed daily through cage‐side observations and weekly by the attending veterinarian during clinical examinations. Activity, posture, behavior, body condition, surgical sites, hydration status, respiration, urine, and feces were monitored using a standardized severity scale to determine the overall health status.

Blood samples for hematology and clinical chemistry were collected on the day of implantation and at study termination. Although these ovine studies demonstrate feasibility, translation to humans will require further evaluation of thrombosis risk, long‐term biocompatibility, and extended performance.

### 2.2. Intravascular Implant Procedure

The intravascular implantation was performed on Day 0. Following administration of analgesia, animals were anesthetized, intubated, and maintained isoflurane. Standard intraoperative monitoring—heart rate, respiratory rate, oxygen saturation (SpO_2_), end‐tidal CO_2_ (ETCO_2_), blood pressure, body temperature, and anesthetic depth—was established prior to the procedure and continued through its duration.

A 5–10‐cm skin incision was made lateral to the jugular vein, and venous access was obtained using the Seldinger technique with an 18G angiography introducer needle and 6‐Fr peel‐away vascular sheath (Cook Medical, Bloomington, Indiana). The sensing lead was advanced into the vein using the introducer kit. Adjacent to the incision, a subcutaneous pocket for the device housing was created via blunt dissection using a 25–30‐mm instrument. The housing was inserted with sufficient slack between it and the sensing lead to minimize the risk of lead migration. The placement of the sensing lead was confirmed using fluoroscopy without contrast. Hemostasis, when needed, was achieved with manual pressure.

The housing was secured to the underlying tissue with nonresorbable 2‐0 nylon sutures. The subcutaneous pocket was closed with absorbable 2‐0 or 3‐0 PDS sutures to eliminate dead space, and the skin was closed with absorbable sutures for both deep tissue and the epidermal layers. Vetbond (3M, St. Paul, Minnesota) was applied to reinforce the skin closure.

Postoperative care included ceftiofur (2.2 mg/kg daily for 3 days), buprenorphine (0.05 mg/kg every 8–12 h), and carprofen (8 mg/kg daily).

### 2.3. Glucose Tolerance Test

Oral glucose tolerance tests (OGTTs) were attempted using 1–2 lbs per animal of sweet feed (sweetened with molasses); however, these OGTTs did not produce blood glucose elevations beyond the normal physiological range for sheep (30–80 mg/dL). This outcome may be due to ruminant physiology, which limits rapid postprandial glucose excursions. Consequently, the IVGTTs were used as the primary metric for device performance.

IVGTTs were conducted in overnight fasted animals. A peripheral venous catheter was used to administer a bolus infusion of 0.5 g/kg of 50% dextrose at approximately monthly intervals throughout the duration of device implantation. During each IVGTT, Glucotrack CBGM measurements were recorded every 2 min for the NFC‐enabled devices and every 10–30 s for the BLE‐enabled devices.

### 2.4. Data Analysis

Microsoft Excel was used for data analysis and graph generation. Outliers within the triplicate Accu‐Chek Guide handheld glucometer measurements were removed using a Dixon′s Q Test (critical value of 0.970; *n* = 3; 95% confidence), and the remaining values were averaged. Each averaged reference glucose value was then paired to the raw, unfiltered Glucotrack sensor measurement collected immediately preceding the reference blood glucose measurement, based on synchronized timestamps.

MARD values for the IVGTT glucose excursions were calculated as the average percent difference between the reference (Accu‐Chek Guide handheld glucometer) and the corresponding unfiltered Glucotrack measurements. Calibration parameters were derived independently for each device at each IVGTT timepoint using a per‐session linear regression. Reference glucometer values greater than two standard deviations from the mean were excluded and omitted from both the retrospective calibration curve and MARD calculations. The Accu‐Chek Guide glucometer is a clinically validated device with performance generally within ±10%–15% of laboratory reference analyzers (e.g., YSI) and is widely used in both clinical and research settings.

### 2.5. Necropsy

**Figure 2:**
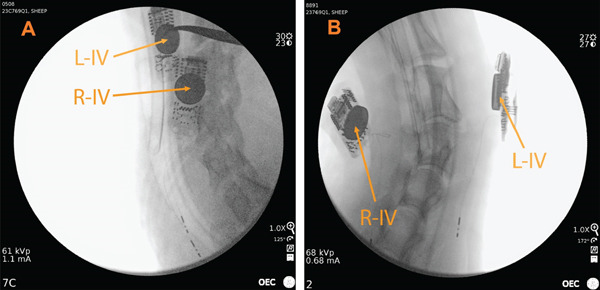
Pretermination imaging example. (A and B) Fluoroscopy images obtained prior to animal termination. Note that R‐IV and L‐IV are right intervascular implant and left intervascular implant, respectively.

Animals were euthanized at their designated study endpoints in accordance with the AVMA Guidelines for Euthanasia of Animals. Prior to euthanasia, fluoroscopic and ultrasound imaging were performed on the animals under isoflurane anesthesia (Figure 2). To minimize postmortem clot formation, heparin (150–200 U/kg) was administered approximately 10 min before euthanasia. Animals were then euthanized using 150 mg/kg of potassium chloride or pentobarbital following sedation with xylazine (0.1–0.2 mg/kg IM), ketamine (2 mg/kg), and diazepam or midazolam (0.05 mg/kg IV). Gross pathological evaluations were subsequently conducted.

### 2.6. Data and Resource Availability

The datasets generated and analyzed contain company‐confidential information and are available from the corresponding author upon reasonable request.

## 3. Results

### 3.1. Animal Health and Clinical/Gross Observations

All animals remained in good health throughout the implantation procedure and the duration of the study. Devices were successfully placed within approximately 20 min, without the need for customized tools. Minor findings attributable to the intravascular devices were noted during gross necropsy; however, none were associated with clinical presentations. No indicators of poor health, stress, or pain were observed at any point. As of December 2025, no clinically significant hematologic or serum chemistry abnormalities suggestive of infection or altered red blood cell parameters were detected at the euthanasia timepoints, although several studies remain ongoing.

Gross examinations of the implant sites showed no evidence of infection, skin erosion, seroma formations that did not resolve, or inflammation. The sensing leads were unremarkable at necropsy, with only minimal fibrin accumulation on their proximal and distal ends. No blood clots, vascular occlusions, or thromboemboli were identified within the jugular veins or in the downstream vasculature. Similarly, no thrombi were observed in the heart, lung, thorax, abdominal organs, or major vessels. In some animals, small areas of the sensing lead appeared to be endothelialized at the venous entry site; however, the leads remained freely floating within the jugular veins. Overall, no major device‐related health complications have been observed in concluded studies.

### 3.2. CBGM Sensitivity

Glucose sensitivities of the CBGMs were determined prior to implantation using a glucose step test (0, 10, 20, 40, 80, and 320 mg/dL dosed at intervals of about 5 min each) on surrogate leads, an example may be seen in Figure S1. In this example, the average sensitivity (slope of the linear regression) of five surrogate leads was 1.13 ± 0.22 nA/mg/dL.

### 3.3. Glucose Excursions

The Glucotrack CBGMs recorded electrochemical measurements every 2 or 10 min for the NFC‐enabled devices and every 10 or 30 s for the BLE‐enabled devices, depending on the experimental setup. IVGTTs were performed approximately monthly (~30, 60, 90, 120, 150, 180, 210, and 240 days postimplantation). Following intravenous bolus injections, glucose levels increased from baseline to hyperglycemic levels (> 300 mg/dL) and returned to baseline within 120 min. The glucose excursion profiles recorded by the CBGMs closely paralleled those measured by the Accu‐Chek Guide handheld glucometer.

**Figure 3:**
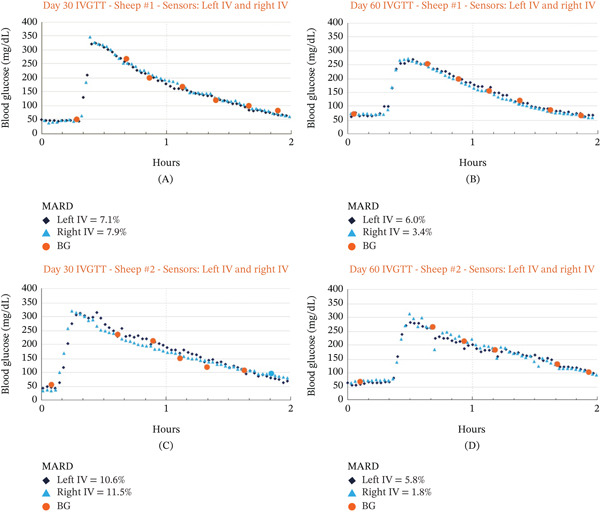
Early example of accuracy of Glucotrack measurements during IVGTT at the 30‐ and 60‐day timepoints. (A and B) Glucotrack measurements (dark blue left; light blue right lines) and Accu‐Chek guide blood glucose measurements (orange circle) for Sheep #1 at the 30‐ and 60‐day timepoints. (C and D) Glucotrack measurements (dark blue left; light blue right lines) and Accu‐Chek Guide blood glucose measurements (orange circle) for Sheep #2 at the 30‐ and 60‐day timepoints.

No lag time corrections or data‐smoothing algorithms were applied to CBGM data. Accu‐Chek Guide measurements greater than two standard deviations from the mean were excluded from the retrospective linear calibration and MARD calculations. The CBGM output (nanoamps) was converted to glucose values (mg/dL) retrospectively using matched Accu‐Chek Guide reference measurements. A simple nonblinded retrospective linear regression was performed for each IVGTT timepoint to correlate CBGM measurements with the reference values. The slope of the linear regression line is the calculated glucose sensitivity (in nA/mg/dL glucose) for each CBGM device. Data points were paired by nearest timestamp; when an exact time alignment was not available, the closest preceding CBGM measurement was used as the matched reference pair. An example of MARD calculations can be seen in Figure 3A–D. Both glucose sensitivity and MARD averages at each timepoint are provided in Table 1.

**Table 1:** Average device sensitivity and MARD across all IVGTT timepoints.

Day	IVGTTs	Average glucose sensitivity (nA/mg/dL)	Average MARD (%)
30	27	1.06 ± 0.394	6.55
60	24	1.07 ± 0.485	8.00
90	13	1.05 ± 0.559	6.30
120	4	1.29 ± 1.055	11.43
150	5	1.24 ± 0.952	3.88
180	3	1.66 ± 0.569	3.37
210	2	1.19 ± 0.135	5.87
240	1	1.09	2.28
Overall	79^a^	1.11^b^	6.84^b^

^a^The number of IVGTTs at each timepoint corresponds to the number of devices contributing data (one IVGTT per device).

^b^Weighted averages were calculated by multiplying the average sensitivity or MARD at each timepoint by the number of IVGTT studies performed at that timepoint, summing these products across all timepoints, and dividing by the total number of IVGTT studies.

### 3.4. Long‐Term Continuous Glucose Monitoring

**Figure 4:**
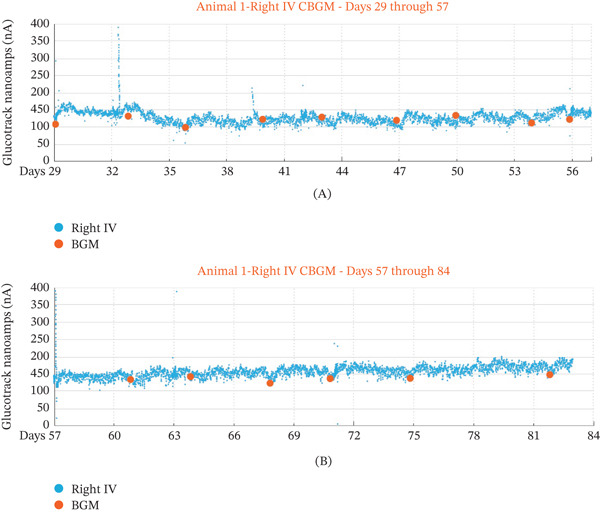
Calibration example. (A and B) Glucotrack measurements (blue circles) and Accu‐Chek blood glucose measurements (orange circle) for 4 weeks before (A) and 4 weeks after (B) calibration at the 60‐day (scheduled on Day 57) IVGTT. Note that the current data spike observed at about Day 32 is due to an IVGTT.

Qualitatively, the raw, unfiltered CBGM measurements had a high signal‐to‐noise ratio with minimal outliers. As an illustrative example, the slope and intercept from a linear regression calibration derived from the 60‐day IVGTT for a CBGM from one animal was retrospectively applied to that sensor′s data 4 weeks before and 4 weeks after the excursion to demonstrate multiweek sensor stability. Retrospectively calibrated glucose measurements of the right‐side CBGM of this animal are shown in Figure 4A,B. With a single calibration, the glucose readings remained stable for the examined 8‐week period, indicating that the CBGM can operate over extended periods with minimal recalibration. In subsequent studies (Animals 2–17), weekly handheld glucometer collections were discontinued to reduce animal handling, and therefore, systematic calibration stability analysis was not performed across all implanted devices.

## 4. Discussion

Here, we present the promising preliminary safety and performance results of Glucotrack′s prototype intravascular CBGM evaluated in an ovine model up to 240 days. Sheep exhibit a stable and narrow euglycemic range (30–80 mg/dL) [14], which enables assessment of device accuracy at glucose levels comparative with human hypoglycemia. Additionally, the elevated glucose concentrations achieved during IVGTTs provided sufficient dynamic range for MARD evaluation. In contrast, OGTTs proved noninformative due to the ruminant physiology, and thus, IVGTTs served as the primary metric for device performance.

MARD is a widely used metric for assessing the accuracy of glucose monitoring devices. An MARD value below 10% is generally considered acceptable for insulin‐dosing decision, with lower MARD values indicating higher accuracy [15]. In our current ovine studies, MARD scores, calculated post hoc from the same measured dataset, were relatively stable across the study (Table 1).

For context, in a separate study, three dogs with diabetes implanted with Eversense XL (predecessor to the currently marketed E3) had an MARD of 24% [16]. In comparison, the Glucotrack CBGM exhibited an average MARD of 6.84% across the duration of the studies. It is important to note that interstitial glucose measurements, such as those obtained from subcutaneous sensors, can be influenced by local blood flow, cellular metabolism, capillary permeability, and hydration [17]. These factors may cause delays or discrepancies between interstitial and intravascular levels, particularly for normal and hyperglycemic individuals [18–20]. In contrast, Glucotrack′s intravascular CBGM displayed no appreciable lag time during glucose excursions, and its measurements remained stable and comparable with the reference device.

Currently, the only commercially available long‐term CGM (as well as integrated CGM or iCGM, which include automated insulin delivery) is the Eversense E3 CGM System (Senseonics). The subcutaneous CGM placed on the upper arm measures interstitial glucose every 5 min up to 180 days with an MARD of 8.5% in humans, although it requires a fingerstick calibration once or twice a day [21]. Modifications to the Eversense E3 system, which extended use up to 365 days, resulted in a comparable MARD of 9.98% [22].

In these initial, proof‐of‐concept in vivo ovine studies, the Glucotrack CBGM demonstrated a strong correlation with reference glucose levels in steady‐state and during dynamic glucose excursions, long‐term stability (up to 240 days), and consistent performance without serious adverse events. The MARD values reported herein fall within or below commonly cited thresholds (< 10%) for CGM systems used in clinical decision‐making. Future work will focus on deployment of production‐grade devices with improved hermeticity and battery life, expanded validation against laboratory reference methods, and first‐in‐human feasibility studies to assess safety, thrombosis risk, and long‐term performance.

### 4.1. Limitations

Several limitations of this study should be noted. The use of prototype devices, characterized by incomplete hermicity and battery‐related limitations, restricted long‐term continuous data collection. Device availability decreased at later timepoints primarily due to battery limitations rather than sensor performance, as reflected in the number of contributing devices at each interval (Table 1). These limitations did not impact IVGTT‐based performance assessments.

Additional limitations include the absence of side‐by‐side comparison between Glucotrack′s CBGM and commercial CGM systems, the lack of continuous reference monitoring outside of IVGTTs, and the reliance on a point‐of‐care handheld glucometer (Accu‐Chek Guide) as the reference standard. Although handheld glucometers provide practical and minimally invasive reference measurements in large‐animal models, laboratory‐based analyzers such as the Yellow Springs Instrument (YSI 2300 Stat Plus) or central clinical chemistry analyzers are considered gold‐standard reference methods and may offer improved analytical accuracy and precision. The use of a handheld glucometer therefore introduces potential measurement variability and represents a limitation of this study. Future studies using production‐grade devices, coupled with continuous or laboratory‐based reference measurements outside of IVGTTs (e.g., YSI or clinical laboratory analyzers, as well as commercial CGMs where appropriate), will be necessary to further strengthen sensitivity assessments, refine MARD calculations, and determine calibration frequency for algorithm development. These subsequent investigations will also be essential for advancing translation to human use through more comprehensive evaluation of thrombosis risk, long‐term biocompatibility, and extended device performance.

## Author Contributions

J.P.T., T.L.R., and M.A.T. were responsible for the design and fabrication of the device electronics. J.G. and M.T. were responsible for the animal model protocol, implantation of devices, glucose tolerance testing procedures, and animal study management. S.T.T. and M.A.T. were responsible for the fabrication of the sensor membranes, in vitro testing, and final device fabrication. J.G., J.P.T., P.V.G., T.L.R., and M.A.T. were responsible for the data analysis. Dr. M.A.T. is the guarantor of this work and, as such, had full access to all the data in the study and takes responsibility for the integrity of the data and the accuracy of the data analysis.

## Funding

No funding was received for this manuscript.

## Disclosure

The initial study comprising data up through 90 days was presented at the ADA 84th Scientific Session in June 2024. All authors contributed and reviewed the completed manuscript.

## Conflicts of Interest

M.A.T., J.P.T., P.V.G., S.T.T., and J.G. are employees of Glucotrack Inc. Both M.T. and T.L.R. are compensated consultants of Glucotrack Inc.

## Supporting information


**Supporting Information** Additional supporting information can be found online in the Supporting Information section. The Supporting Information materials provide additional preimplant data and methodological details. The study performed glucose sensitivity tests on surrogate leads using a standard glucose step method, yielding preimplant glucose sensitivity results. In vivo data were retrieved twice a week from the device′s internal memory using an NFC reader and processed for configuration and evaluation. The NFC data downloads were limited in order to reduce the stress on the animals caused by the handling and the amount of restraining required. The BLE‐enabled devices automatically uploaded data to the mobile‐based cloud, which allowed for real‐time monitoring on the noncalibrated data. Further details on glucose tolerance test methodologies are also included.

## Data Availability

The data that support the findings of this study are available from the corresponding author upon reasonable request.
