# Supplementary material for: In Vivo Evaluation of a Novel Long‐Term Intravascular Implantable Continuous Blood Glucose Monitor in an Ovine Model: A Glucotrack Inc. Investigation
Source: J Diabetes Res. 2026 Apr 25;2026:8838426. doi: 10.1155/jdr/8838426 (PMC13110273; doi:10.1155/jdr/8838426)
Supplement: Supplementary file 1 — Supporting Information Additional supporting information can be found online in the Supporting Information section. The Supporting Information materials provide additional preimplant data and methodological details. The study performed glucose sensitivity tests on surrogate leads using a standard glucose step method, yielding preimplant glucose sensitivity results. In vivo data were retrieved twice a week from the device′s internal memory using an NFC reader and processed for configuration and evaluation. The NFC data downloads were limited in order to reduce the stress on the animals caused by the handling and the amount of restraining required. The BLE‐enabled devices automatically uploaded data to the mobile‐based cloud, which allowed for real‐time monitoring on the noncalibrated data. Further details on glucose tolerance test methodologies are also included. [file JDR-2026-8838426-s001.docx]

**Supplemental**

*Supplemental Figure 1. Pre-implant glucose calibration.*

Glucose sensitivity was performed on surrogate leads using a standard glucose step test of seven glucose levels (0, 10, 20, 40, 80, and 320 mg/dL). In the figure provided, the dosing of a concentrated stock glucose solution occurred at about 620, 920, 1220, 1520, 1820 and 2120 seconds to achieve 10, 20, 40, 80, and 320 mg/dL concentrations respectively.


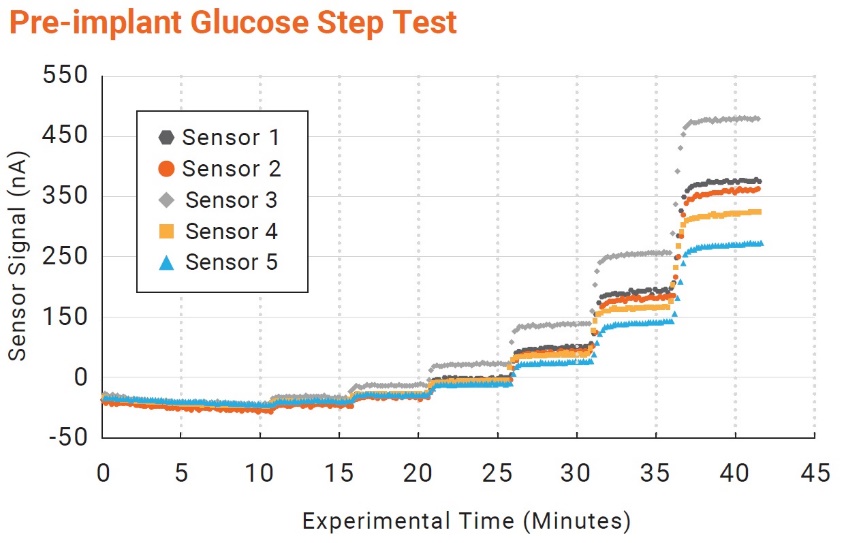


*Supplemental Methods*

An in-study data collection check was performed by retrieving the data from the internal memory stored in the device housing using an NFC reader (ISC.MR102-USB, FEIG Electronics Inc.) and antenna (ANT340/240) twice a week. Data snapshots were processed by ST25PC-NFC software (STMicroelectronics) to configure device settings and evaluate the most recent data records.

Oral glucose tolerance test (OGTT) was sweet feed with added molasses given 1-2 lbs per animal for the first eleven days after surgery then once a week for the rest of the study. The oral glucose tolerance test (OGTT) did not elicit a blood glucose level change higher than the typical range for sheep (30-80 mg/dL). These results are not unexpected since the ruminant ovine model used was non-diabetic. Unfortunately, IVGTT data for the 90-day timepoint were not collected due to device power loss around days 84-88.
